# Development and psychometric evaluation of the Premarital Sexual Behavior Assessment Scale for Young Women (PSAS-YW): an exploratory mixed method study

**DOI:** 10.1186/1742-4755-11-43

**Published:** 2014-06-13

**Authors:** Azam Rahmani, Effat Merghati-Khoei, Lida Moghadam-Banaem, Ebrahim Hajizadeh, Mostafa Hamdieh, Ali Montazeri

**Affiliations:** 1 Department of Midwifery & Reproductive Health, Faculty of Medical Sciences, Tarbiat Modares University, Tehran, Iran; 2 Iranian National Center of Addiction Studies (INCAS); the Risk Behaviour Institution, Tehran University of Medical Sciences, Tehran, Iran; 3 Department of Biostatistics, Faculty of Medical Sciences, Tarbiat Modares University, Tehran, Iran; 4 Infertility and Reproductive Health Research Center (IRHRC), Shahid Beheshti University of Medical Sciences, Tehran, Iran; 5 Mental Health Research Group, Health Metrics Research Center, Iranian Institute for Health Sciences Research, ACECR, Tehran, Iran

## Abstract

**Background:**

Premarital sexual behaviors are important issue for women’s health. The present study was designed to develop and examine the psychometric properties of a scale in order to identify young women who are at greater risk of premarital sexual behavior.

**Method:**

This was an exploratory mixed method investigation. Indeed, the study was conducted in two phases. In the first phase, qualitative methods (focus group discussion and individual interview) were applied to generate items and develop the questionnaire. In the second phase, psychometric properties (validity and reliability) of the questionnaire were assessed.

**Results:**

In the first phase an item pool containing 53 statements related to premarital sexual behavior was generated. In the second phase item reduction was applied and the final version of the questionnaire containing 26 items was developed. The psychometric properties of this final version were assessed and the results showed that the instrument has a good structure, and reliability. The results from exploratory factory analysis indicated a 5-factor solution for the instrument that jointly accounted for the 57.4% of variance observed. The Cronbach’s alpha coefficient for the instrument was found to be 0.87.

**Conclusion:**

This study provided a valid and reliable scale to identify premarital sexual behavior in young women. Assessment of premarital sexual behavior might help to improve women’s sexual abstinence.

## Background

Risky sexual behavior is commonly defined as behavior that increases one’s risk of contracting sexually transmitted infections and experiencing unintended pregnancies. They include having sex at an early age, having multiple sexual partners, having sex while under the influence of alcohol or drugs, and unprotected sexual behaviors [1,2]. Indeed having sex at an early age or premarital sexual behavior could harm adolescents’ overall health in general and their sexual health in particular [3]. The association between premarital sexual activities and the risk of acquiring sexually transmitted infections or increased risk of unprotected sexual behaviors are well documented [4,5]. In addition there is evidence that adolescents who engage in premarital sexual activities may not experience the same quality marital relationship and stability as the married people who abstained from premarital sexual activities [6]. Thus to prevent young adolescents from adverse outcomes of premarital sexual behaviors, sexual abstinence has been recommended [7,8].

However, similar to other societies the age of marriage has risen in Iran [9]. The mean age at marriage was 19.7 years for women in 1976, compared to 23.4 in 2011. Also, number of women aged 15–29 years who had never married was estimated to be more than 5.5 million in 2011. Consequently, the gap between puberty and marriage (the only legal way permitting young people to experience their first sexual encounter) has considerably increased. Thus one might believe that Iranian youth are remaining sexually virgin or innocent during their bachelor lives. However, contrary to expectation, a recent study reported that the prevalence of premarital relationships is rising among Iranian young people [10]. It has been estimated that 27.7% of Iranian young people experience sexual intercourse at age fifteen. While the report alarms the likelihood of rising risky sexual behaviors [11], there is no comprehensive sex education targeting youth in Iran. However, in the Iranian contexts, sexual health education and services have a variety of challenges and obstacles for unmarried youth [12]. Sexual health services such as sexual counseling or family planning are planned only for married people and do not target youth, as alleged by advocates and scholars [8,9]. Another concern is the outcomes of premarital sexual relationships that have more devastating outcomes for women comparing to men. If these relationships do not lead to marriage, the emotional and social consequences tend to be greater for women than for men due to the importance of virginity for young women’s marriage prospects [10]. Therefore, identifying young women who are at greater risk of premarital sexual behavior is an important issue in conservative societies such as Iran.

Most sexuality instruments are focus on sexual function [13-16], quality of sexual life [17], intimacy and sexual satisfaction [18-20] and sexual abuse [21]. Fewer instruments exist about premarital sexual behaviors. For instance, the Self-Efficacy to Refuse Sexual Behavior Scale (RSB) [20] aims to assess individuals’ ability to refuse sexual behavior when they did not want to have sex with someone. However, this questionnaire does not contain items on other aspects of sexual behavior including knowledge; attitude; and protective behaviors. As such some scholars recommended that for studying sexuality in young people we need to develop instruments that are be able to predict risky behaviors [21,22]. Thus, the purpose of the present study was to develop and examine the psychometric properties of a newly developed scale in order to identify young women who are at greater risk of premarital sexual behavior and perhaps indicate areas for implementing interventions to improve sexual abstinence among young women.

## Methods

The present study was an exploratory mixed method investigation. Indeed, it has been conducted in two phases. For the first phase, a qualitative approach was applied in order to generate items and develop the questionnaire. In the second phase, psychometric properties of the questionnaire were assessed.

### Phase 1: Item generation and scale development

#### Research design

In this qualitative inquiry, a two-step qualitative approach, using focus group discussions and individual interviews, was used to develop a premarital sexual behavior in young women.

#### Participants and data collection

We recruited a group of young women (n = 63) aged 18–34, who volunteered to participate in the study. We employed focus group discussions as the primary means of data gathering. The sessions were facilitated by defining sexual behaviors, and using a semi-structured inventory that began with the open ended questions: ‘How is the sexual life for young Iranian women’. Afterward, based on the responses obtained from the participants, subsequent questions built upon the discussion. Although Iran’s constitution is based on Islamic law and government policies are guided by Shari’a and Islamic principles, new generation, in particular, may not believe speaking openly about sexuality is against religion or cultural norms [23]. However, the participants with different levels of religiosity challenged each other’s viewpoints in learning and experiencing sexuality. We documented our analytic ideas by memo writing. The focus group discussions enabled the investigators to identify the potential informants for individual interviews. Those women who had premarital sexual activity were identified during FGDs and invited for individual interviews (4 out of 63 participants). Although, women who had premarital sexual experience did not speak openly in the discussion, we identified these women when they spoke about their beliefs. For example, some of them believed that premarital sexual experience is a natural experience and every woman, similar to a man, could enjoy it. Also, in order to have access to other young women with premarital sexual experience, snowball sampling was used and additional 8 young females were identified for individual interviews. Sampling was continued with maximum variation to yield greater transferability of data and saturation [24]. To achieve maximum variations, informants were selected from different age groups, different socioeconomic backgrounds, having various types of sexual experiences, and being as high and low level religiosity characters. Deep individual interviews provided a situation for us to speak about their beliefs and religious practices and thus could be able to identify the level of their religiosity. Participants had varying educational level. Most were living in dormitories. A few of them were living with their families at the time of interviews. Six focus group discussions were held and twelve participants were interviewed individually. Data saturation was achieved after 5 focus group discussions and 10 individual interviews.

#### Data analysis

Inspired by Graneheim and Lundman’s approach we employed qualitative content analysis. In this approach ‘the most suitable unit of analysis is whole interviews or observational protocols’ [25]. Data analysis commenced during the data collection. Each focus group discussion and individual interviews was transcribed verbatim and analyzed before the next focus group discussion or interview took place. We achieved thorough comprehension of the data by reading and re-reading. In the next step, the units of meanings were extracted from the statements. Data analysis proceeded using line-by-line coding; codes were created during repeated discussions between researchers. Categories and themes were created based on the codes with similar meanings. In total, 4 key themes and 23 subthemes emerged as important factors relating to premarital sexual behaviors. The framework has been shown in Table 1. Finally, an item pool containing 53 statements was generated and has been used for psychometric evaluation.

**Table 1:** Themes and sub-themes identified by the focus groups and individual interviews (phase 1)

**Theme**	**Sub-themes**
**Individual predisposing factors**	
	Fear of losing boyfriend
	Sexual curiosity
	Discounting the values
	Lack of perceived threat
	Inconsistency between one’s believes and social norms
	Seeking freedom
	Motivational beliefs
	Lack of self-efficacy
**Social predisposing factors**	
	Different sexual motivation in male and female
	Lack of social support
	Reduction in marriage opportunity
	Modern media
	Generation gap
	Family dysfunction
	Gender-based sexual expectation
	Conservative context and lack of sexuality education
**Individual protective factors**	
	Perceived susceptibility
	Negative emotions regarding premarital sexual behavior
	Protective beliefs
	Self-management
**Social protective factors**	
	Observational learning
	Family support and supervision
	Importance of virginity in singlehood

### Phase 2: Psychometric evaluation of the Premarital Sexual Behavior Assessment Scale in Young Women

#### The questionnaire

As indicated earlier, the first draft of the questionnaire was developed on the basis of the findings of the focus group discussions and individual interviews. The pre-final draft of the Premarital Sexual Behavior Assessment Scale for Young Women (PSAS-YW) contained 53 items and each item is rated on a five-point response scale (completely agree to completely disagree).

#### Sampling

A sample of young women aged 18–34 were recruited from university campus, dormitories, and parks. We recruited all single young women regardless of their sexual performance. Women who did not intend to respond to the questionnaire carefully were excluded. Sample size was estimated based on the number of items in the questionnaire multiplying by 10 as recommended [26]. Thus a sample of 260 young women was thought. However, since there was a risk for incomplete questionnaires, 270 individuals were approached. Demographic characteristics of the young women included recoding of age, education, and occupational status.

### Statistical analysis

Several statistical methods were used to analyze the data:

1. Validity: we assessed content, face, and construct validity of the Premarital Sexual Behavior Assessment Scale for Young Women (PSAS-YW) as follows:

Content validity: Qualitative and quantitative content validity were applied. An expert panel consisting of a team of investigators specialized in sexuality and psychometric assessed the content validity of the questionnaire. In the qualitative phase, they evaluated wording, grammar, item allocation, and scaling of the questionnaire. In the quantitative phase, the content validity index (CVI) and the content validity ratio (CVR), were calculated. Clarity, simplicity, and relevancy of each item is assessed by CVI evaluation [27,28]. In order to assess the CVI, we used a Likert-type, ordinal scale with four possible responses. The responses contain a rating from 1 = not relevant, not simple and not clear to 4 = very relevant, very simple and very clear. CVI was calculated as the proportion of items that received a rating of 3 or 4 by the experts [29]. The essentiality of items was tested by calculating the CVR. For calculating the CVR, the experts rated each item as essential, useful but not essential, or not essential [30].

Face validity: qualitative and quantitative methods were applied to evaluate face validity. In the qualitative phase, 10 young women were asked to evaluate the questionnaire and indicate if they felt difficulty, or ambiguity in responding to the questionnaire. In the quantitative phase, the impact score (frequency × Importance) was calculated to indicate the percentage of young women who identified the item as important or quite important. Items were considered appropriate if they had an impact score equal to or greater than 1.5 (which corresponds to a mean frequency of 50% and a mean importance of 3 on the 5-point Likert scale) [31].

Construct validity: Exploratory factor analysis (EFA) was performed to determine the underlying constructs of the questionnaire. A principle component analysis (PCA) with varimax rotation was applied and the factor loading equal to or greater than 0.4 was considered acceptable [26].

2. Reliability: the Cronbach’s alpha coefficient was calculated to assess the internal consistency of the questionnaire. Values equal to or greater than 0.70 were considered satisfactory [32]. In addition, in order to assess the questionnaire’s stability, test-retest reliability was conducted to estimate the intraclass correlation coefficient (ICC). Twenty participants completed the questionnaire twice with two-week intervals. ICC values of 0.40 or higher were considered satisfactory (r ≥ 0.81-1.0 as excellent, 0.61- 0.80 very good, 0.41-0.60 good, 0.21-0.40 fair, and 0.0-0.20 poor) [33].

### Ethics

Approval to conduct the study was granted by the Ethics Committee of the Faculty of Medicine of Tarbiat Modares University, Tehran, Iran. The participants were informed that participation in the study was voluntary, their confidentiality would be maintained, and none of the participants would be identified in any publications arising from the study. Informed written consent was obtained from the participants.

## Results

### Participants

In all 270 young women were approached. Of these the data for 265 individuals was complete and included in the analysis. The mean age of participants was 25.5 ± 3.8 years; and most (72%) were living in dormitories. The characteristics of the study participants are shown in Table 2.

**Table 2:** Demographic characteristics of the study sample (phase 2, n = 265)

		**Number**	**Percent**
**Age (years)**			
	18-20	33	13
	21-30	207	77
	31-34	25	10
	Mean (SD)	25.5 (3.8)	
	Range	18-34	
**Educational status**			
	High School Diploma	21	8.7
	Bachelor	77	28.7
	Master	124	37.2
	PhD	43	25.4
**Occupational status**			
	Student	199	75
	Housewife	9	3.4
	Employed	57	21.6

### Validity

#### Content validity

In the quantitative content validity phase, items with CVR and CVI less than 62 and 80, respectively, were omitted (24 items). In the qualitative phase, other criteria such as grammar, wording, and item allocation were edited according to the experts’ opinions. For example, the sentence “I believe having sexual relationship is a sign of open-mindedness” changed to “sexual relationship is a sign of open-mindedness” or “Being with a man makes me relax” changed to “Being with a man is relaxing for me”.

#### Face validity

Impact score was calculated to examine quantitative face validity. Impact score had ranged from 1.2 to 5. Therefore, 3 items were omitted and the pre-final version of the questionnaire containing 26 items was preserved for the next steps of psychometric assessment. In the qualitative face validity, participants stated that they have had no problems in reading and understanding the items.

#### Construct validity

Exploratory factor analysis (EFA) was used to evaluate construct validity. The Kaiser-Meyer-Olkin (KMO) and Bartlett’s test illustrated that the data was proper for factor analysis (KMO index = 0.87, χ^2^ = 2136.62, P < 0.001). Principal component analysis with varimax rotation identified five factors with eigenvalues greater than 1 and factor loading equal to or greater than 0.4; accounting for 57.4% of variance observed. The factor loadings were as follows:

● Factor 1with 8 items including item 1, 2, 3, 4, 7, 8, 9, and 13 (Motivating beliefs).

● Factor 2 with 7 items including item 10, 17, 19, 20, 21, 25, and 26 (Performance).

● Factor 3 with 5 items including item11, 12, 22, 23, and 24 (Facilitators).

● Factor 4 with 4 items including item 14, 15, 16, and 18 (Inhibiting factors).

● Factor 5 with 2 items including item 5 and 6 (Virginity pledge). The results are shown in Table 3.

**Table 3:** Exploratory factory analysis of the PSAS-YW^*^

**Item**	**Factor 1**	**Factor 2**	**Factor 3**	**Factor 4**	**Factor 5**
**1**	**.740**	.272	.092	.126	.097
**2**	**.845**	.157	.126	.005	.075
**3**	**.680**	.125	.243	-.060	.123
**4**	**.572**	.131	.001	.079	-.139
**7**	**.645**	.243	.230	-.045	-.008
**8**	**.745**	.356	.121	.060	.130
**9**	**.687**	.257	.167	.048	.148
**13**	**.748**	-.132	.025	.218	.031
**10**	.352	**.462**	.350	-.043	-.047
**17**	.448	**.550**	-.046	-.090	-.001
**19**	-.023	**.741**	.048	-.016	.298
**20**	.488	**.562**	.141	.079	.007
**21**	.205	**.603**	.310	-.035	.225
**25**	.385	**.562**	.212	.000	-.119
**26**	.170	**.773**	.089	-.040	-.102
**11**	.371	-.013	**.535**	-.027	.118
**12**	.417	.245	**.481**	-.039	-.279
**22**	.133	.108	**.764**	.026	-.080
**23**	.089	.146	**.764**	-.064	.266
**24**	-.005	.396	**.502**	-.005	-.168
**14**	.037	.007	.053	**.839**	.031
**15**	.174	-.076	-.030	**.851**	-.148
**16**	.150	-.061	-.063	**.756**	.103
**18**	-.122	.047	-.041	**.522**	.315
**5**	.607	.062	.207	.077	**.476**
**6**	.207	.078	-.002	.145	**.781**

^*^Figures in bold are related to factors loaded equal to or greater than 0.4.

### Reliability

Internal consistency was used to evaluate reliability. The Cronbach’s alpha coefficient for the questionnaire was 0.87 and for the subscales ranged from 0.70 to 0.88, well above acceptable thresholds. In addition, the ICC for the questionnaire was found to be 0.85 (good to excellent), lending support to the stability of the questionnaire.

## Discussion

The results from this study showed that the PSAS-YW is valid and reliable. In fact, the CVI and the CVR indicated a reasonable content validity. In addition, the Cronbach’s alpha coefficient and intraclass correlation coefficient were acceptable and indicated good reliability and stability for the questionnaire. The final 26-item PSAS-YW contained five subscales (motivating beliefs, performance, facilitators, inhibiting factors, and virginity pledge). Items included in ‘motivating beliefs’ and ‘facilitators’ subscales reflected conditions and beliefs that might encourage young women to experience premarital sexual behaviors. The ‘Inhibiting factors’ and ‘virginity pledge’ subscales were designed to reflect factors impeding premarital sexual behavior including issues related to personal concern and factors related to social norms and values. Items in the ‘performance’ subscale referred to behaviors, habits, and life styles, which young women select to engage or not to engage in premarital sexual behaviors.

There are few instruments for assessing premarital sexual behavior. For instance, recently Dunsmore and collegues developed the Sexual Abstinence Motivation Scale-SAMS [34]. The SAMS contains 41 items incorporated into 8 subscales (commitment to self-schema, risk of disappointing authority figures, fear/apprehension of the sexual experience, fear of physical consequences, value of virginity, reputation regret, no opportunity/not important, and manipulation). They have reported satisfactory psychometric properties for the scale. Although most domains of the SAMS (fear of the sexual experience, fear of physical consequences, value of virginity, and reputation regret) were similar to the items or domains of our instrument (the PSAS-YW), some domains such as commitment to self-schema and risk of disappointing authority figure, were different. Perhaps these differences might be related to the differences in various social and cultural norms in the two studies. However, the present study was conducted only among women while the former study has been conducted on both sexes.

The Sexual Abstinence Behavior Scale (SABS) is another instrument for measuring premarital sexual behavior that mainly focuses on abstinence. Psychometric evaluation of the SABS showed satisfactory results for its reliability and validity. The scale has been introduced for advanced practice nurses to promote sexual abstinence of their young adolescent patients [35]. The main problem with this instrument is the fact that its application is very limited and it is not applicable for other health-care professional groups.

We found that religious beliefs were important inhibiting factors for premarital sexual experiences. Similar concept was evident in the literature where Goggin et al. [36] introduced a new instrument namely the Sexual Risk Behavior-related God Locus of Control measure for Adolescents (SexGLOC-A). They reported on development of a questionnaire that measures the effect of ‘God control beliefs’ on sexual risk behaviors. For instance, some beliefs included the perception that God is [or is not] actively helping them to delay sexual experience, refuse engagement in risky sexual behaviors, or limit the number of their sexual partners.

The current study had some limitations. Sexuality is a relatively private subject with varying degrees of social, cultural, religious, moral and legal norms and constraints [37]. Research on premarital sexuality faces an additional difficulty in Iran because sexual behaviors before marriage are unacceptable and forbidden by law [10]. In the present study, most people preferred not to speak about their sexual behaviors. Thus, the main investigator tried to decrease this limitation by establishing rapport and trust. Also, a major limitation of our study was the fact that we have not tested convergent or known-groups validity and that the majority of the participants were students (75%) or employed (21.6%). It seems that in a further testing of the measure: urban/rural; level of education; religion should also be taken into account. The sample was constituted mostly of people with high level of education and living in campus, which can constitute an important confound factor for conclusions about the understanding and consistency of the questionnaire should it be used with different cohorts. Finally further examination, especially confirmatory factor analysis for the PSAS-YW, is suggested.

In summary, one of the millennium development goals is to prevent sexually transmitted disease, including HIV [38]. Additionally, it is recommended that goal of any program is to be measured correctly [39]. We thought developing a measure for assessing premarital sexual behavior in order to improve premarital sexual abstinence might cover this goal and help to improve women’s sexual health.

## Conclusion

The findings from this study provided preliminary evidence for psychometric properties of the Premarital Sexual Behavior Assessment Scale for Young Women (PSAS-YW). Further studies in various populations of young women are needed to establish stronger psychometric properties for the questionnaire.

## Competing interests

Authors declare that they have no competing interests.

## Authors’ contribution

AR was the main investigator, designed the study, collected the data and wrote the first draft. EMK and LMB were the study supervisors. EH contributed to the statistics and was the study consultant. AM was the study consultant, critically reviewed the manuscript, responded to reviewers’ comments and provided the final draft. All authors read and approved the final manuscript.
